# Micromechanical Response of Crystalline Phases in Alternate Cementitious Materials using 3-Dimensional X-ray Techniques

**DOI:** 10.1038/s41598-019-54724-8

**Published:** 2019-12-05

**Authors:** Sriramya D. Nair, Kelly E. Nygren, Darren C. Pagan

**Affiliations:** 1 School of Civil and Environmental Engineering, Cornell University, 527 College Ave, Ithaca, NY 14853 USA; 2 Cornell High Energy Synchrotron Source, 161 Synchrotron Dr, Ithaca, NY 14853 USA

**Keywords:** Civil engineering, Mechanical properties, Ceramics

## Abstract

Cementitious materials are complex composites that exhibit significant spatial heterogeneity in their chemical composition and micromechanical response. Modern 3-dimensional characterization techniques using X-rays from synchrotron light sources, such as micro-computed tomography (*μ*CT) and far-field high-energy diffraction microscopy (ff-HEDM), are now capable of probing this micromechanical heterogeneity. In this work, the above mentioned techniques are used to understand the varying micromechanical response of crystalline phases (cubic iron oxide and *α*-quartz) inherently present within an alkali-activated fly ash (AAF) during *in*-*situ* confined compression. A subset of the crystals probed using ff-HEDM are registered with the tomographic reconstructions and tracked through the applied loads, highlighting the combination of *μ*CT and ff-HEDM as a means to examine both elastic strain in the crystalline particles (and by extension local stress response) and plastic strain in the matrix. In this study, significant differences in the load carrying behaviors of the crystalline phases were observed wherein the cubic iron oxide crystals laterally expanded during the confined compression test, while the *α*-quartz particles laterally contracted and at the final load step, shed load likely due to failure in the surrounding matrix. Finally, the two characterization techniques are discussed in terms of both advantages and associated challenges for analysis of multi-phase cementitious materials.

## Introduction

By quantity, concrete is the world’s most prevalent structural material and its production comes with a sizable carbon footprint: production of one ton of portland cement generates 0.9 tons of CO_2_^1^. In 2017, 4.1 billion tons of cement were produced worldwide resulting in approximately 5% of the global CO_2_ emissions^2^. As such, any advances that reduce the amount of portland cement produced, either through replacement with more environmentally-friendly alternatives or more judicious usage, can provide major ecological benefits. However, before alternative cementitious materials (ACM) are adopted or new design parameters implemented, the response of the material under its expected environmental conditions must be well understood. Over the past decade, several laboratory-scale tests have been conducted to probe the behavior of ACMs^3,4^, but significant gaps still exist between our understanding of the connections between microstructure and macroscale properties.

The example ACM studied herein, alkali-activated fly ash (AAF), falls under a class of alkali-activated binders that form inorganic polymers^5^. This class of materials is generally produced by activating an alumino-silicate powder such as fly ash, blast furnace slag, or metakaolin, with an alkaline solution such as sodium/potassium hydroxide occasionally mixed with sodium/potassium silicates^6^. Apart from being low cost and environmentally friendly^7^, these materials are receiving increasing attention as a replacement for portland cement-based concretes because of their: resistance to alkali aggregate reaction^8,9^, freeze-thaw durability^10,11^, sulfate attack^12,13^, corrosion resistance^14^, improved early age shrinkage^15^, less susceptibility to carbonation^16,17^, ability to encapsulate nuclear waste^18^, improved compatibility with oil well drilling fluids^19^ and good adhesion and binding to multiple surfaces including metallic substrates^20^.

Although these materials are promising, their use in the industry has been limited. A path forward for accelerating the adoption of ACMs requires a better understanding of how the micro structure of a cementitious material ultimately dictates its macroscale performance^21–24^. Ideally, characterization of a micro structure could lead directly to prediction of performance, precluding the need for exhaustive macroscale testing to find materials suitable for use. Efforts have been placed to understand dissolution kinetics^5^, geopolymerization process^5^ and degree of hydration^25^ in order to improve prediction models. One of the greatest identified challenges in the modeling of cement-based materials is accounting for the heterogeneous spatial distribution and properties of the various phases in the cement paste. To date, the primary tool for characterizing the spatial heterogeneity of mechanical response at lower length scales has been nanoindentation^22,26–28^. While providing excellent spatial resolution of elastic moduli across phases, the highly localized nature of the measurement prevents it from being able to explore interactions between phases and how these interactions change with deformation. In this work, we demonstrate how methods such as 3-dimensional (3-D) X-ray absorption and diffraction-based characterization techniques can be used to quantify the micromechanical response of crystalline phases. In ACMs, the response of these phases is influenced by the surrounding matrix, and understanding these interactions brings the field one step closer to the rapid adoption and effective use of by-products and waste materials.

The X-ray techniques used to study the AAF in this work are micro-computed tomography (*μ*CT) and the far-field variant of high-energy diffraction microscopy (ff-HEDM). The use of *μ*CT^29^ to characterize damage induced in cementitious materials is well established for studying the evolution of void and crack growth under load^30–33^, corrosion-induced cracking^34^, alkali-silica reaction^35,36^, sulfate attack^37,38^, autogenous healing^39^, leaching^40^, and freeze-thaw cracking^41^. X-ray diffraction has been used to study cement hydration and geopolymerization kinetics^42–48^ using synchrotron light sources, however the application of 3-D diffraction techniques, such as ff-HEDM, to study micromechanical behavior of cementitious materials is significantly less well established. The ff-HEDM technique is capable of quantifying the orientation, centroid, and elastic strain state of individual crystals as a sample is deforming *in*-*situ*. A combination of *μ*CT and ff-HEDM applied to probing the deformation of a model portland cement paste embedded with ~100 *μ*m pure single crystal *α*-quartz, which served as distributed micromechanical measurement points, is the only [known] study to date^49^. The model system, however, avoided many data analysis challenges associated with tracking crystalline phases that are inherently present in most cementitious materials.

In general, cementitious materials are composed of multiple crystalline phases with a wide distribution of crystal sizes, producing diffraction signals ranging from distinct diffraction peaks to continuous Debye-Scherrer rings. The ff-HEDM technique relies on the ability to track diffraction peaks from individual crystals generally in the 10 *μ*m to 500 *μ*m range. Other X-ray scattering from fine crystalline phases (crystal size <10 *μ*m) or amorphous phases are also captured on the detector, but are not the focus of the current study. Herein, we will explore how ff-HEDM can be used to analyze the elastic strains of phases with crystals of size and number density conducive to producing distinct single crystal diffraction peaks on the detector and subsequently the distribution of load within these crystals.

The structure of the paper is as follows. *μ*CT and diffraction based strain data are provided in the results section. The results are discussed, with special focus placed on the challenges of data reduction and possible means to extract more from the data, followed by a summary. In the Methods section, the composition of the fly ash used and the sample preparation method are provided. Next, the experimental and data processing procedures are outlined. In this work, compressive normal strains and stresses will be defined as positive.

## Methods

### Materials and sample design

**Figure 1:**
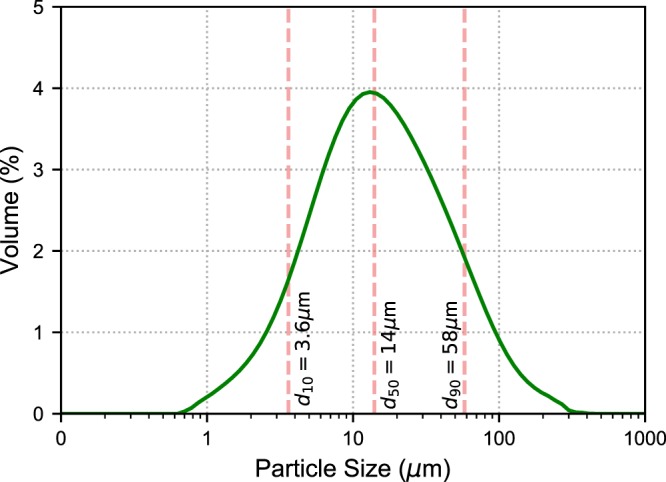
Particle size distribution of the fly ash studied in this work.

A reclaimed, thermally-beneficiated class F fly ash^50^ from a coal power plant in Maryland was used in this study. The oxide composition was provided by the manufacturer (SEFA group) and can be viewed in Table 1. The fly ash is composed of ~70% aluminosilicates by weight and more than 20% iron oxide, with minor amounts of calcium oxide and magnesium oxide. Fly ash in general is composed of both crystalline and amorphous phases with a wide distribution of particle sizes. The particle size distribution of the raw fly ash particles was obtained using Malvern Mastersizer 2000 with a wet dispersion unit and can be viewed in Fig. 1. The d_50_ of the fly ash is 14 *μ*m, indicating that 50% of the particles by volume are larger than 14 *μ*m.

**Table 1:** Oxide analysis of fly ash used in this work.

	weight (%)
CaO	7.5
SiO_2_	45.7
Al_2_O_3_	24.0
Fe_2_O_3_	20.7
MgO	0.8
Na_2_O+0.658K_2_O	1.7
SO_3_	0.5

**Figure 2:**
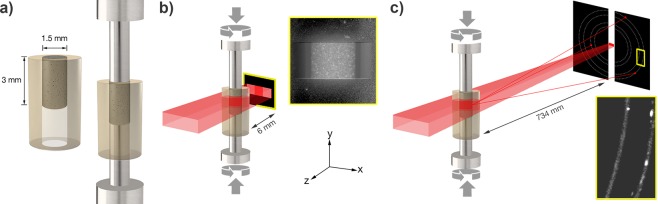
Schematic of the (**a**) AAF specimen within a PEEK tube; (**b**) experimental geometry for the micro-computed tomography (*μ*CT) measurements along with the coordinate system for the sample reference frame. Inset: Example transmission radiograph from the AAF specimen used for the 3-D tomographic reconstructions; (**c**) experimental geometry for the far-field high-energy diffraction microscopy measurements (ff-HEDM). Inset: Magnified section of example diffracted intensity from the AAF specimen.

The AAF sample was prepared by mixing the fly ash described above with 8 M sodium hydroxide solution at a solution-to-fly ash ratio of 0.485 (by weight) based on preliminary tests for optimum strength and workability. 8 M sodium hydroxide solution was prepared by weighing reagent grade sodium hydroxide pellets and dissolving them in ultrapure water (resistivity of 18 MΩ-cm). The solution was set aside for at least 24 hours to allow for dissipation of heat. The AAF slurry was mixed in a 15 ml centrifuge tube using a vortex mixer for 1 minute. A vortex mixer was used instead of a high shear mixer^51^ because of the high viscosity of the slurry. After mixing, the slurry was injected into a 1.59 mm $(\tfrac{1}{16}^{\prime\prime} )\,{\rm{ID}}$, 3.18 mm $(\tfrac{1}{8}^{\prime\prime} )\,{\rm{OD}}$, 5 mm long polyether ether ketone (PEEK) tube (c.f. Fig. 2a) using a blunt #18 gauge needle. PEEK was chosen for its resistance to degradation in the presence of unreacted sodium hydroxide solution. A 3 mm tall volume of slurry was injected into the tube, providing a 2 mm gap at the bottom of the PEEK tube, which was used for guiding the sample onto the bottom loading platen. The current sample design was formulated in an attempt to follow common practice for compressive strength testing (cylinders in ratio of 1:2 for diameter:height), along with ensuring the cylindrical samples were centralized on the loading platen and in the path of the beam.

The AAF slurry contained within the PEEK tube was cured in a high humidity environment at 76 °C (170 °F) for 10 days. Typically for mixtures with low calcium fly ashes, it has been reported in literature that curing temperatures in the range of 40°–100 °C yield higher early strengths^3^. Prior to this work, extensive research was conducted to understand the behavior of this AAF for oil well cementing, a bottom hole temperature of 76 °C is typical. Furthermore, the age was chosen to satisfy time constraints associated with synchrotron use and so that the results could be compared to existing results^52^ in the 7–14 day age range. Details about the mechanical properties of this AAF slurry in comparison to ordinary portland cement slurry can be found elsewhere^52^. Care was taken to ensure both the top and bottom surface of the sample were flat and parallel to each other. This ensures that the sample will remain vertical and the load will be transmitted uniformly along the loading axis.

### Experimental description

The AAF specimen was deformed under confined uniaxial compression at the F2 station of the Cornell High Energy Synchrotron Source (CHESS) with confinement provided by the previously described PEEK tube. Loading was performed using the second generation of the Rotation and Axial Motion System (RAMS2). RAMS2 is a screw-driven uniaxial loading system that allows for 360° of rotation without impeding X-ray measurements as a sample is loaded *in*-*situ*^53^. The compressive load was applied by Inconel718 (nickel-based alloy) platens with a diameter of $\tfrac{1}{16}^{\prime\prime}$, sized to slide through the PEEK tubing without friction (c.f. Fig. 2a). X-ray data was collected prior to loading and at six intermittent load steps as the sample was compressed. At each load step, a peak load was applied and then the sample was allowed to relax while holding the compression platens in position. Once the load had stabilized, X-ray measurements were performed.

A schematic of the experimental geometry is given in Fig. 2. In the given geometry, the incoming X-ray beam travels in the −***z*** direction, while the sample is loaded and rotated during measurement along the ***y*** axis. For the X-ray measurements, the sample was illuminated by a 41.991 keV (*λ* = 0.296 Å) X-ray box beam that was 1.1 mm tall and 2.5 mm wide. In order to probe the entire volume of the specimen, three separate measurements were performed at heights spaced 1 mm apart in the specimen (providing 50 *μ*m of overlap between layers). For each of the three volumes and at each of the load increments, the sample was rotated 180° to obtain transmission radiographs (Fig. 2b). The radiographs were obtained at 0.1° rotation increments (totaling 1800 radiographs), using a Retiga 4000DC camera coupled to a LuAG:Ce scintillator with a 5× lens (1.48 *μ*m effective pixel pitch) placed 6 mm from the sample. An example radiograph is shown in the inset of Fig. 2b. The scintillator was then moved out of the path and the sample was rotated 360°, capturing diffraction patterns (Fig. 2c) at 0.25° rotational increments (totaling 1440 patterns) using two Dexela 2923 detectors (74.8 *μ*m effective pixel pitch, 3888 × 3072 pixels) that were positioned 734 mm away from the sample. The detector distance and the mounted angle with respect to the sample location were calibrated using CeO_2_ standard reference material, followed by a pre-calibrated multiruby crystal with known parameters. A magnified view of part of the diffraction image obtained for the AAF specimen is presented in the inset of Fig. 2c, showing a diffuse diffraction ring with regions of intense, isolated diffraction peaks.

### Data processing

3-D tomographic reconstructions were generated from the raw radiographs using custom Python scripts based on a GPU implementation of the Simultaneous Iterative Reconstruction Technique (SIRT) algorithm within the ASTRA-toolbox 1.8.3^54,55^ through TomoPY 1.1.2^56^. The spacial resolution of the *μ*CT reconstructions are in the order of 1.45 *μ*m. The centroids, orientations, and elastic strain tensors within crystals of different phases were found from the diffraction data using the HEXRD software package 5.26^57^. The ff-HEDM processing algorithm consists of ‘indexing’ and fitting routines. Indexing is the process of associating individual diffraction peaks with crystals in the illuminated volume. First, for a given crystal type and lattice orientation, the positions and angles at which diffraction peaks will appear are simulated. If a prescribed set of these peaks are found in the measured experimental data (quantified in the output as ‘completeness’), the measured diffraction peaks are then associated with a crystal now determined to be in the illuminated volume. The process is repeated for a discrete set of possible lattice orientations for each crystal type of interest to index as many crystals present as possible. Once diffraction peaks have been associated with crystals present, an optimization procedure is used to refine the lattice orientation, centroid, and elastic strain tensor ***ε***^*E*^. This procedure is repeated at each load step to monitor the evolution of elastic strain state of the indexed crystals. For measurements performed at the F2 beamline, experimental uncertainties in elastic strain tensor components are ~10^−4^ and 0.05° for lattice orientations^58^. More complete details of the solution algorithm can be found in^57^.

The assembling of data (both *μ*CT and ff-HEDM) from different volumes along the sample height and the registration of data from different measurements were performed using custom scripts developed in Python. The registration of different data types was performed by matching centroids of crystals found using the ff-HEDM method with the nearest crystals found in the tomographic reconstruction.

## Results

### Macroscopic response and tomographic reconstructions

**Figure 3:**
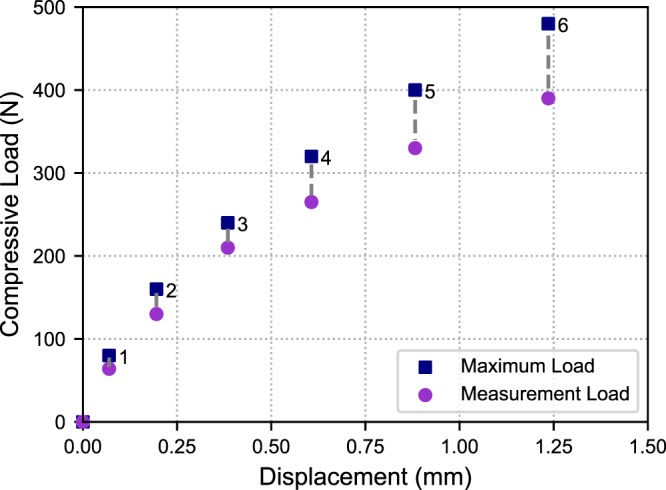
Macroscopic compressive load versus displacement values at which X-ray measurements were made. Both the maximum load applied and the stress after relaxation (where X-ray measurements were performed) are shown.

The macroscopic mechanical response at each load step where X-ray measurements were performed is plotted in Fig. 3 as the macroscopic compressive load vs total displacement. The crosshead was held fixed after each load increment throughout the duration of the X-ray measurements. After each load increment, the stress in the sample relaxed, so both the maximum load applied and the load after relaxation (‘Measurement Load’) are plotted in Fig. 3.

**Figure 4:**
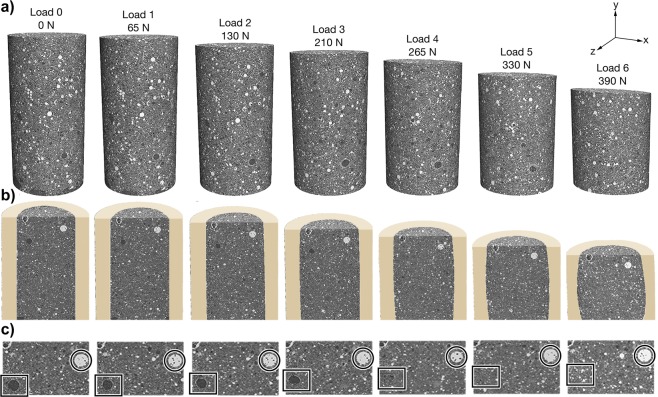
(**a**) Tomographic reconstructions of 10-day old AAF specimen prepared at 76 °C (170 °F) under compression at various load steps. At Load 0 the sample was 3 mm tall and 1.5 mm in diameter. (**b**) Cross sectional view along with the PEEK tube to highlight the bulge in the sample at the later load steps. (**c**) A magnified view to showcase the ability to track air voids and large particles. Open source softwares TomoPY 1.1.2^56^ along with ASTRA-toolbox 1.8.3^54,55^ were used for reconstructing the radiographs and ParaView 5.5^73^ was used for 3D visualization.

Tomographic reconstructions generated from the raw radiographs at each load step are shown in Fig. 4a for the entire sample volume. *In*-*situ* tomographic reconstructions provide valuable information regarding the microstructural neighborhood, influencing the measured micromechanical loading in the crystalline phases. The grey scale values of the reconstruction are proportional to the local density and, as such, are useful for identifying various phases. Also, with the high spatial resolution (≈2 *μ*m), a much clearer understanding of the shape and motions of phases and voids/cracks can be attained. Evolution of micromechanical loads in crystalline phases measured using ff-HEDM while monitoring void collapse and crack growth may be of particular value in the future for predicting mechanical response.

In Fig. 4, a cylindrical mask was applied to the tomographic reconstruction to remove the PEEK tube from visibility. The sample height is shown to decrease with increasing applied load, as expected. This significant decrease is shown to be accompanied by lateral bulging of the specimen at later load steps, Fig. 4b, and the collapse of the largest voids in the sample, Fig. 4c. In the *μ*CT measurements, the various intensities in the gray scale of the images are related to the material density (related to composition) at each pixel, where the darkest regions correspond to voids and the brightest regions correspond to the densest phase. The box in Fig. 4c (of a region near the top surface in Fig. 4b) highlights the tracking of a specific void which begins to collapse at Load 3 and is no longer present at the later load steps. The circle in 4c shows the ability to track a particle and obtain it’s new position at each applied load. Cracks large enough to be identified with the available resolution were not observed during loading.

### Micromechanical response of crystalline phases

While numerous crystalline phases were present in the specimen, isolated diffraction peaks from two of the phases were analyzed using the ff-HEDM technique. An example of these distinct diffraction peaks is shown in the inset of Fig. 2c. The first crystalline phase identified was cubic iron oxide with an average lattice parameter *a*_0_ of 8.387 Å. The second crystalline phase was trigonal *α*-quartz with average lattice parameters *a*_0_ and *c*_0_ of 4.914 Å and 5.406 Å respectively. The ff-HEDM method was used to determine crystal orientation, centroid, and elastic strain state from the diffraction data obtained at each of the load steps.

At Load 0, ~250 cubic iron oxide crystals and ~80 *α*-quartz crystals were identified (indexed) and crystal parameters found with high confidence. For this material, high confidence consists of finding more than 90% of predicted diffraction peaks in the diffraction data and optimizing crystal parameters that maintain a solution consistency (related to Pearson’s ${\chi }^{2}$ test statistic) of less than 0.05 with the measured diffraction peak positions.

#### Combined spatial and micromechanical response of cubic iron oxide

**Figure 5:**
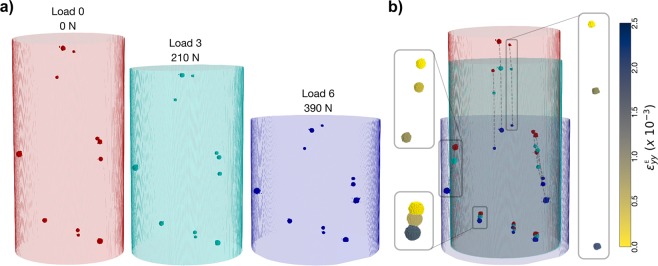
(**a**) Using custom python scripts, cubic iron oxide spheres were isolated from the full *μ*CT reconstruction and are shown for Loads 0, 3 and 6. (**b**) The movement of the grains are tracked across the three load steps and the insets show the corresponding elastic strains along the loading direction. An opensource 3D visualization software, ParaView 5.5^73^ was used to view the reconstructions.

Based on the centroids obtained from ff-HEDM, a few of the cubic iron oxide crystals were identified and isolated from the 3-D *μ*CT reconstructions and registered with the corresponding elastic strain data measured using ff-HEDM. The cubic iron oxide particles were easily isolated from the surrounding matrix due to the good visual contract between the particles and the matrix. Such isolation was challenging for the *α*-quartz crystals due to similarities in density (and related pixel value in the reconstruction) with the surrounding matrix. Isolated tomographic reconstruction of the cubic iron oxide particles at Loads 0, 3, and 6 are shown in Fig. 5a. These reconstructions show the motion of these particles with increasing compressive load. These motions can also be viewed in the dotted paths of Fig. 5b. The bottom half of the specimen shows significantly smaller displacements in comparison to particles in the upper half of the sample because the bottom platen remains stationary during testing. The translation of these particles with increasing load can be ascribed to the *total* strain of the material (both elastic and plastic strain). The ff-HEDM strain tensor measurement registered to these particles, on the other hand, provides only a measure of the *elastic* strain response. The insets of Fig. 5b colors the particles according to the compressive elastic strain component along the loading direction ${\varepsilon }_{yy}^{E}$ measured using ff-HEDM. This strain component is shown to increase in magnitude for each of the example particles as a function of load step. The combination of the two measurement types provides a more complete picture of the micromechanical response: total particle motion from *μ*CT provides information about the total strain state and ff-HEDM provides the elastic strain information.

#### Elastic mechanical response of cubic iron oxide and *α*-quartz

**Figure 6:**
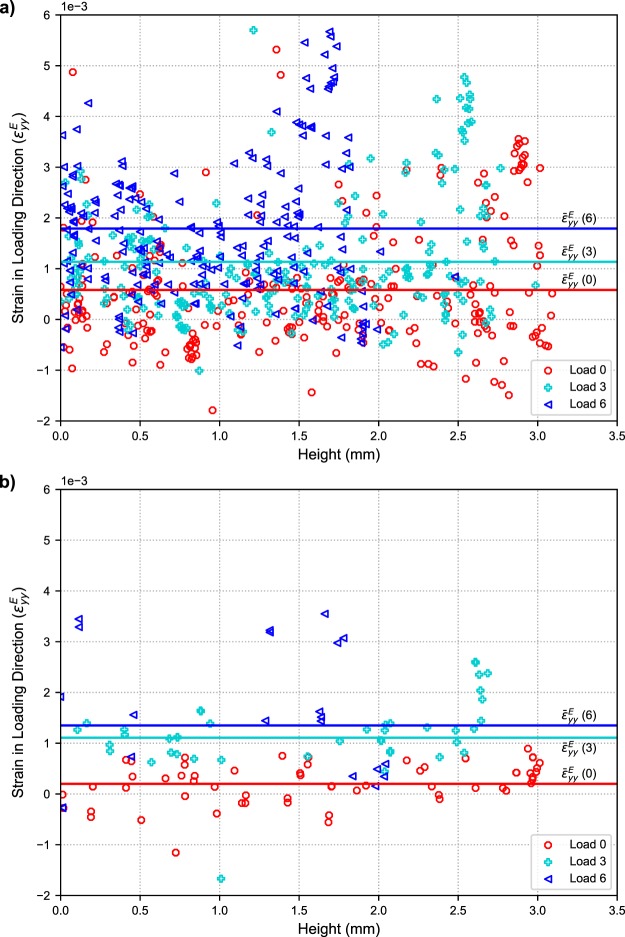
(**a**) Distribution of elastic strain values ${\varepsilon }_{yy}^{E}$ versus vertical position (height) in the AAF specimen for (**a**) cubic iron oxide crystals and (**b**) *α*-quartz crystals. Data with a high confidence index are shown. Note: In this figure compressive normal strains and stresses are positive.

**Figure 7:**
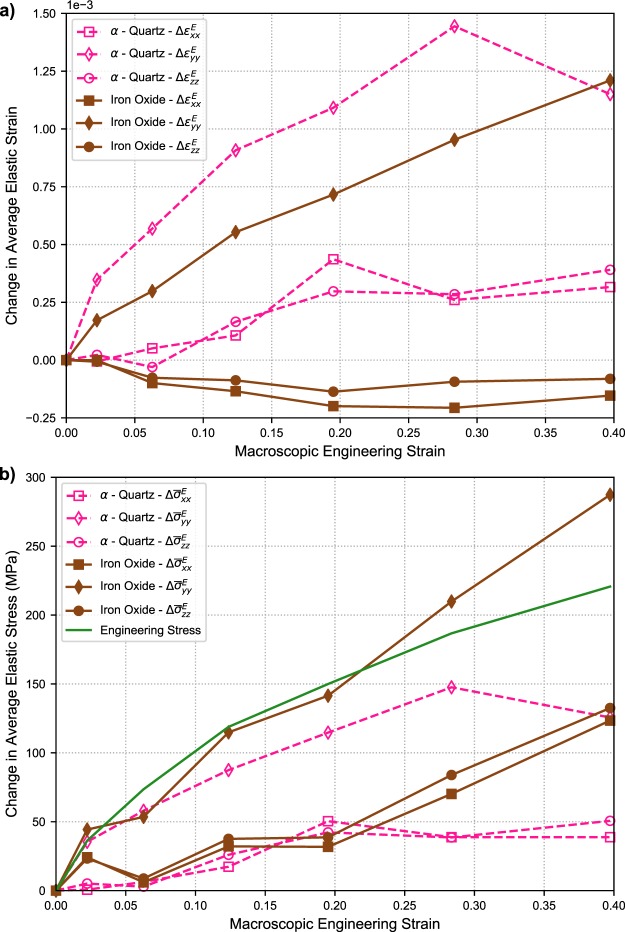
(**a**) Evolution of the average change in normal elastic strain components with increasing macroscopic strain in the cubic iron oxide and *α*-quartz crystals. (**b**) Evolution of the average change in normal stress components with increasing macroscopic strain in the cubic iron oxide and *α*-quartz crystals. Note: In this figure compressive normal strains and stresses are positive.

Before comparing the elastic mechanical response of the two indexed crystalline phases, it is important to note that while this paper presents only the normal components of the strain tensor for analysis, all six components of the elastic strain tensor (and by extension elastic stress tensor) were calculated using the ff-HEDM technique. Figure 6 shows the elastic strain component in the loading direction, ${\varepsilon }_{yy}^{E}$, as a function of position along loading direction at Loads 0, 3, and 6 for (a) cubic iron oxide and (b) *α*–quartz crystals. At Load 0, in the case of the cubic iron oxide phase, there is a large distribution in the strain ${\varepsilon }_{yy}^{E}$ values (−2 × 10^−3^ to 4 × 10^−3^), much greater than the per component uncertainty of ~10^−4^ which has been previously determined for this technique^58^. This large variation in strain across the cubic iron oxide crystals can be attributed to variation in lattice parameter (calculated to be in the range of 8.344–8.404 Å) due to substitution of Fe atoms by Ca, Al, Mg, Mn or Ti during coal combustion^47,59^ and possible local variations of shrinkage stresses. Comparing elastic strains in the loading direction at various load steps, it can be seen that on average the compressive elastic strains in both phases increased as the macroscopic load applied to the specimen was increased (refer to lines plotted across Fig. 6). It should be noted that with the ff-HEDM analysis method used, more than half of the *α*-quartz crystals initially indexed with high confidence could not be tracked at Load 6, likely due to the beginning of crystal fracture which will be discussed later. To compare average elastic strain behavior across the two phases, the change (Δ) in the average strains for the three normal strain components ${\varepsilon }_{xx}^{E}$, ${\varepsilon }_{yy}^{E}$, and ${\varepsilon }_{zz}^{E}$ are shown in Fig. 7a. To calculate these values, the strain components from each identified crystal in the sample frame are averaged. The change in this average value from Load 0 are then determined. For $\Delta {\varepsilon }_{yy}^{E}$, the values within the *α*-quartz were generally larger than the cubic iron oxide until Load 6. The $\Delta {\varepsilon }_{xx}^{E}$ and $\Delta {\varepsilon }_{zz}^{E}$ values within each phase are generally similar as expected from the symmetry of the loading and the specimen. The primary difference occurs in the components transverse to the loading direction, wherein the *α*-quartz is generally contracting (positive change in elastic strain), while the cubic iron oxide is expanding (negative change in elastic strain). This is possibly due to the interactions of these crystals with the matrix and the confinement provided by the PEEK tube leading to non-uniaxial loading conditions. Based on current findings from the tomographic reconstructions and micromechanical data, sample designs that provide less constraint are being considered for future experiments.

In order to examine the differences in load transmitted through the two phases, the anisotropic form of Hooke’s Law

1 $${\boldsymbol{\sigma }}={\mathbb{C}}:{{\boldsymbol{\varepsilon }}}^{E},$$

where ***σ*** is stress and ${\mathbb{C}}$ is the fourth order stiffness tensor, was used to calculate stresses from the elastic strain tensors of the crystals. Since the lattices within the crystals can be oriented in any direction with respect to the loading axis, determination of stress in each crystal was simplified by performing the calculation in a frame fixed to the lattice basis vectors of each crystal (crystal frame). The process for calculating the average change in stress was to use the elastic strains in the crystal frame, evaluate Hooke’s law to determine the current grain stress, transform the stresses back to the sample frame, and lastly, average the stress tensor components from all high confidence crystals. Changes in the average stress are calculated from Load 0. The single crystal moduli for the cubic iron oxide used^60^ were (in GPa): ${C}_{11}=260.5$, ${C}_{12}=148.3$, and ${C}_{44}=63.3$, while the *α*-quartz moduli used^61^ were: ${C}_{11}=87.2$, ${C}_{12}=6.6$, ${C}_{13}=11.9$, ${C}_{14}=-\,17.2$, ${C}_{33}=105.8$, ${C}_{44}=57.2$, and ${C}_{66}=40.4$. The average changes in stress with increasing macroscopic strain can be seen in Fig. 7b. The macroscopic engineering stress calculated from applied load is also shown in Fig. 7b for a general reference of the applied stress magnitude.

The change in average stresses in the two phases along the loading direction are of the same order of magnitude as the applied macroscopic engineering stress. Although it should be made clear that there are many more constitutive phases in the bulk that support the applied load, the crystal stresses calculated are found to be reasonable for the system. Due to the sample being confined in the PEEK tube, the stresses in the two phases are generally not uniaxial and are compressive for all normal stress components. From the changes in stress in Fig. 7b, it can be seen that the increase in stresses in the cubic iron oxide phase along the loading direction are larger than the *α*-quartz phase. The increase in transverse stresses in the cubic iron oxide phases are also larger than in the *α*-quartz phase. We also see that there appears to be an inflection to higher load in Δ*σ*_*yy*_ in the cubic iron oxide phase and an inflection to lower load in the *α*-quartz phase, while noting that many *α*-quartz crystals could no longer be tracked at Load 6.

## Discussion

In this work, the micromechanical response of the alternative cementitious material, alkali-activated fly ash, was quantified *in*-*situ* using both imaging (*μ*CT) and spatially resolved diffraction (ff-HEDM) techniques. ff-HEDM provided the ability to locally quantify the elastic strain, and by extension calculate the stress, in crystalline particles spread throughout the sample, while *μ*CT provided information about the rigid displacement of these crystals through the matrix due to plastic deformation.

The iron oxide and *α*-quartz crystals are a natural component of the AAF studied here (rather than an additional material added solely as a probe) and play a role in the micromechanical response of the AAF. While the stresses determined from ff-HEDM data are the average from within these crystals, it is important to remember that the stress state is dictated not only by the local elastic properties (stiffness of crystal), but also the local boundary conditions governed by the surrounding matrix^62^: a change to the surrounding matrix will alter the stress state in the crystals. As such, these stresses provide an important window to gaining understanding about phase interactions in the AAF *in*-*situ*, and subsequently predicting the micromechanical response. In addition, the interfaces between stiff inclusions and a more compliant surrounding matrix are often found to nucleation points or easy paths for traversal for cracks^63^, and ultimately drivers for failure.

Using the elastic strain and stress data shown in the results section, some of the spatial heterogeneities and interactions of mechanical response at the *μ*m to mm length scale can be explored. In Fig. 7a, the effect of crystal composition on the mechanical (strain) response can be observed. For most of the test, the changes in strains along the loading direction ($\Delta {\varepsilon }_{yy}^{E}$) in the *α*-quartz were higher than the cubic iron oxide due to *α*-quartz’s lower stiffness (the inflection at the final load step is likely due to inelastic events in the *α*-quartz). The translation from elastic strain to stress using Hooke’s law also provides interesting insights into load partitioning. Even with only probing two of the crystalline phases, this study captured distinct mechanical responses, with the *α*-quartz contracting and the cubic iron oxide phase expanding on average transverse to the loading direction.

The average change in stresses in the crystals also provides complimentary information about load partitioning. While, the average changes in strain in the cubic iron oxide crystals along the loading directions were generally lower than the *α*-quartz, the average changes in stress ($\Delta {\sigma }_{yy}^{E}$) were higher. In the results, it was mentioned that there appears to be a redistribution of stress along the loading direction at Load 6 between the cubic iron oxide and *α*-quartz phases (an increase and decrease in $\Delta {\sigma }_{yy}^{E}$). At the same time, there was a significant decrease in the number of *α*-quartz crystals that could be tracked using ff-HEDM. A loss of the ability to track crystals in ff-HEDM is usually due to a degradation of diffraction peak quality, namely decreases of intensity and broadening caused by an increase in defect content. The loss of tracking of the *α*-quartz crystals and the load redistribution are likely related. As the *α*-quartz crystals begin to develop internal fracture networks, regions within each crystal become misoriented, diffraction peaks broaden, and the crystal loses its ability to support increased load. Similar behavior has been observed during the compression of a loose, granular collection of *α*-quartz crystals^64^. As the macroscopic load is still increasing, other constituent phases, such as the cubic iron oxide, must support a relatively larger percentage of the applied load.

For both the crystalline phases, the changes in transverse stresses ($\Delta {\sigma }_{xx}^{E}$ and $\Delta {\sigma }_{zz}^{E}$) were increasingly compressive in load, but the transverse stresses stop increasing in the *α*-quartz at Load 4. Conversely, the rate of change of transverse stresses in the cubic iron oxide appears to increase from Load 4 onwards. While the average phase response may provide a general overview of how load is partitioned among the different phases, the stress responses of individual crystals is strongly related to the neighboring matrix response. It is important to remember that these crystals were embedded as part of a surrounding matrix that strained significantly more than the relatively hard crystalline phases. The stress and strain responses are not just due to the phase stiffnesses, but rather a combination of crystal stiffness and surrounding matrix. ff-HEDM is providing a means to probe these interactions.

To explore the impact and response of surrounding matrix on individual crystals, the ff-HEDM techniques can, with some effort, be paired with computed tomography techniques. Figure 5 shows an example of isolating a few crystals and tracking their spatial movement, which can be related to an associated total strain. With volumetric image correlations, there is an opportunity to track the total strain of a material by simply tracking all the particles, and voids, as they move through each volume. By pairing this information with the local elastic stress response from a crystal, the response of the surrounding matrix (such as fracture), and reciprocal effects such as void compaction on local stress state, can be directly probed. It is the dynamic response of the crystals and matrix within their neighborhoods that is essential to capturing the impact of spatial heterogeneity on material properties. However, in complex material systems, identifying regions of interest and isolating individual crystals, let alone thousands of crystals of varying phases, and their response has many associated challenges. Adding to this complexity is the limited resolution currently available to automate the identification and isolation of crystals. Smoothing algorithms and robust segmentation schemes that can be implemented on noisy and large data sets need to be developed.

While the initial results for probing micromechanical response in cement pastes are promising, there are difficulties that need to be addressed with regard to processing and interpreting the diffraction data for cementitious materials in comparison to metallic alloy systems for which the ff-HEDM technique was developed. Cement pastes made from portland cement or recycled materials such as the AAF studied here are not only composed of multiple phases but also have significant heterogeneity within the composition of each phase^65,66^. The ability to measure an *absolute* lattice strain and the related stress (rather than a change) is intimately tied to knowing the initial local lattice parameters of the individual crystals. The wide spread of initial strains (at Load 0) in Fig. 6 may be an artifact of differences in unstrained lattice parameters. However, we also note that the crystals may indeed have residual strains due to shrinkage during the curing process. Separating the effects of composition difference and true residual strains is a challenge for all diffraction based stress measurements^67^ and is particularly acute for the study of concrete. Furthermore, the conversion of lattice strains to stresses using Hooke’s law is based on chosen single crystal moduli for a pure phase. Further work is warranted to explore the effect of compositional changes on the single crystal elastic moduli.

The ff-HEDM technique used in this work is only suitable for extracting information from isolated diffraction peaks. For multi-phase samples such as the AAF studied here, scattered X-rays from both amorphous and fine-grained crystalline phases are also detected. It should be highlighted that the scattering from these phases, although not a focus this study, can be analyzed to gain more information about load partitioning, albeit without the ability to locate the position of scattering in the illuminated volume and register the loading data with a tomographic reconstruction. The broad scattering of the amorphous phases can be used to determine correlation lengths between atoms which will expand or contract with applied load^68,69^. While the fine-grained crystalline phases can be analyzed using strain pole figure methods to study the crystal lattice orientation dependence of load partitioning^70–72^. However, the challenge lies in the ability to deconvolve the overlapping scattering from different phases. New image processing methodologies will need to be developed and adopted before these analyses can be performed simultaneously. Although if made possible, a more complete picture of the micromechanical response of all phases present can be reconstructed and used to inform modeling efforts. Analyzing complex scattering patterns from other cementitious systems, including portland cement, are currently underway.

## Summary

To aid the development of micromechanical models of complex cementitious systems, the combination of two high-energy X-ray 3-D characterization techniques has been demonstrated. Far-field high-energy diffraction microscopy measurements provided the ability to probe the partitioning of load within different crystalline phases (cubic iron oxide and *α*-quartz) present in an alkali activated fly ash specimen, while the combination of *μ*CT and ff-HEDM demonstrated the ability to visualize the local strains and understand their effect on the surrounding matrix. Such information is vital for modeling the deformation of cementitious materials and also to probe the effect of heterogeneity on the micromechanical response.
